# New Application for Old Ulcers

**Published:** 1874-09

**Authors:** 


A NEW APPLICATION FOR OLD ULCERS is always in order, and no apology is necessary for giving the following, which was very highly lauded by a writer in the *Lancet* a year or two ago, and has been made the subject of a communication to one of the French journals recently. It consists of flour, four ounces; tragacanth, one-half ounce; powdered acacia, one ounce; powdered chalk, one drachm; one egg; water, one pint. Put these materials in a stew-pan, and bring them nearly to boiling; then allow the mixture to cool, and add water to form the whole into a thin paste, which may be applied twice daily, without washing off. The ulcer to be covered with a soft rag. The mixture should not be allowed to become sour.—*Philadelphia Medical Times—June.*

